# A Case of Megaspleen With Micrographism

**DOI:** 10.7759/cureus.29270

**Published:** 2022-09-17

**Authors:** Subrahmanyan VR, Deepu V Joy, Sweta Sahu, Anagha SK, Abhishek LNU, Vempati Roopeessh, Prerna Chandra, Vishal Venugopal, Sourav Bansal, Rishman Tandi

**Affiliations:** 1 Internal Medicine Pediatrics, Armed Forces Medical College, Pune, IND; 2 Internal Medicine, 167 Military Hospital, Pathankot, IND; 3 Internal Medicine, JJM Medical College, Davanagere, IND; 4 Internal Medicine, Government Medical College, Thiruvananthapuram, IND; 5 Medicine, Government Medical College, Amritsar, IND; 6 Internal Medicine, Gandhi Medical College and Hospital, Hyderabad, IND; 7 Internal Medicine, Deccan College of Medical Sciences, Hyderabad, IND; 8 Internal Medicine, Bhaarath Medical College and Hospital, Chennai, IND

**Keywords:** accumulation of copper, zinc therapy, fulminant hepatic failure, ceruloplasmin transferrin, wilsons disease

## Abstract

Wilson disease is an inherited disorder of copper metabolism with an autosomal recessive inheritance pattern (hepatolenticular degeneration). In this case, a 13-year-old child was seen with overall exhaustion, sporadic abdominal discomfort, and shrinking handwriting during the previous 12 months. On clinical examination there was hepatosplenomegaly. Routine blood work revealed anemia, leukopenia, thrombocytopenia, elevated total and indirect bilirubin, alkaline phosphatase and transaminitis. Serum ceruloplasmin is decreased, urine copper excretion is increased. Slit lamp examination revealed Kayser-Fleischer rings in both eyes. Ultrasonography (USG) abdomen confirmed hepatosplenomegaly, coarse echotexture of the liver. MRI Brain revealed the bilateral and diffuse and symmetric hyperintensity of caudate and lentiform nuclei, which are consistent with the neuro-parenchymal changes of Wilson’s disease. We report this case due to its rare incidence and atypical presentation and to highlight the importance of clinical examination in reaching the diagnosis.

## Introduction

Hepatolenticular degeneration, another name for Wilson's disease, is a rare autosomal recessive inborn error of copper metabolism that causes decreased excretion of copper and defective incorporation into ceruloplasmin, which results in accumulation of copper in the liver, brain, eyes, and other tissues [1]. Hepatic or neurologic symptoms are more frequently observed in young people than psychiatric symptoms. Tremor, poor coordination, choreoathetosis, and dystonia are examples of neurologic symptoms. Depression, neurotic behaviours, personality changes, and intellectual decline are examples of psychiatric symptoms [2]. One case of Wilson's illness is present for every 30,000 live births worldwide [3]. Other than India, the incidence across Asia ranged from 33 to 68 per 100,000. Although Wilson's disease is infrequent, if it is not recognised and treated, it can result in major harm and even death. Although the diagnosis is easily missed, if it is made early on, there are effective treatments that can stop or reverse many of the disorder's symptoms. Wilson's disease is typically identified by the presence of specific neurological symptoms, the presence of Kayser-Fleischer rings when viewed under a slit lamp, the reduction of serum ceruloplasmin levels, the elevation of hepatic and urine copper levels, and decreased serum levels of ceruloplasmin [4]. We report an adolescent presented with soft symptoms on multiple emergency room visits and missed obvious clinical signs of a major systemic illness.

## Case presentation

**Figure 1:**
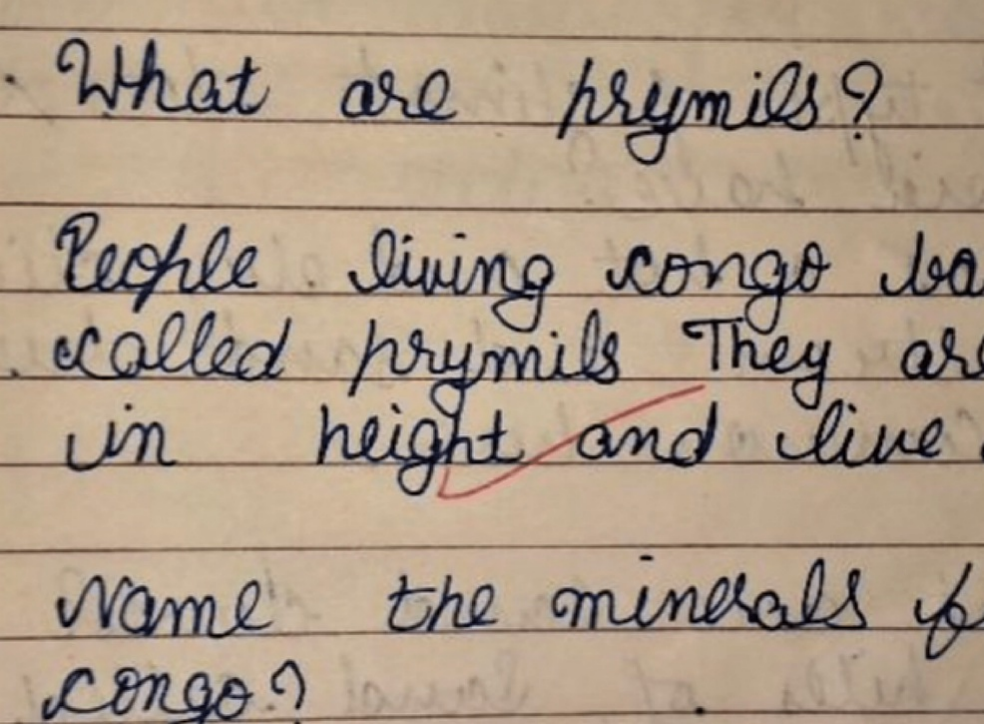
Handwriting of the child one year prior

**Figure 2:**
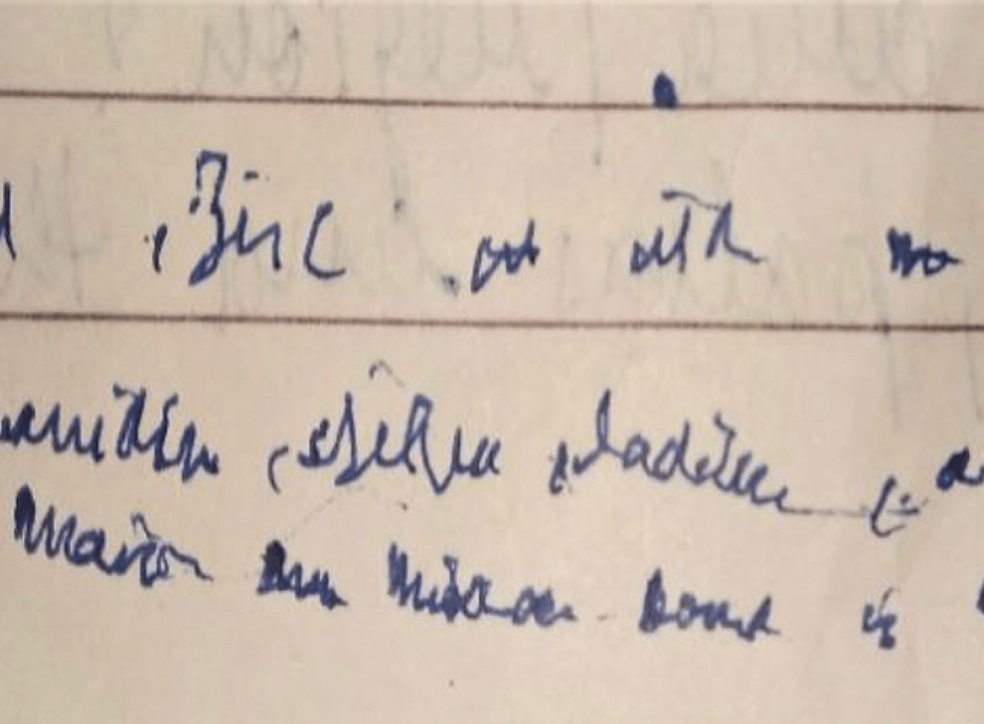
Handwriting at present shows micrographia

A 13-year-old boy third born of non-consanguineous marriage from Northern India was brought with history of generalised fatigue and occasional abdominal discomfort of two to three months duration. Subsequent detailed history revealed progressive diminution in the size of his handwriting affecting his school performance and excessive drooling of saliva during day and night. No history suggestive of any change in behaviour, speech, memory, cranial nerve deficits, motor weakness, sensory deficits, ataxia or abnormal movements. There was no history suggesting any constitutional symptoms of fever, weight loss, joint pain or rash. He was immunized as per universal immunization programme and had no family history of neurological illness. Clinical examination revealed that the child was conscious and his vital parameters were normal. On neurological examination, higher mental functions and speech of the patient were normal. But there was significant change in handwriting in form of micrographia. Handwriting of the child a year ago was neat and very clear (Figure 1). However, at presentation, his handwriting was abnormally tiny and was difficult to read (Figure 2). There was no signs of cranial, motor, sensory deficits and cerebellar involvement clinically. 

On per abdominal examination, Hackett’s grade 3 splenomegaly with firm consistency and enlarged liver (span 11cm) with firm consistency, smooth surface with sharp margins. Patient was admitted with working diagnosis of chronic liver disease with central nervous system involvement (extrapyramidal). Various laboratory investigations were conducted (Table 1).

**Table 1:** Laboratory findings of the patient Hb- Hemoglobin RBC- Red Blood Cell RDW- Red Cell Distribution Width TLC- Total Leucocyte Count PBS- Peripheral Blood Smear AST- Aspartate Transaminase ALT- Alanine Transaminase ALP: Alkaline Phosphatase PT- Prothrombin Time INR- International Normalised Ratio HIV- Human Immunodeficiency Virus HCV- Hepatitis C Virus HBsAg: Hepatitis B surface Antigen

LABORATORY INVESTIGATIONS	PATIENT’S VALUES
HEMATOLOGY	Hb-10.8g/dl (Adult males: 13-16g/dl Adult Females: 12.1-15.1g/dl Children: 11.5-15.5g/dl New born: 13.5-24g/dl) RBC count- 3.99mil/mm^3^ (Adult males: 4.7-6.1mil/mm3 Adult Females: 4.2-5.4mil/mm3 Children: 4-5.5mil/mm3 New born: 4.8-7.1mil/mm3) RDW- 18% (Adult males: 11.8-14.5% Adult females: 12.2-16.1% Children: 12.3-14.1% New born: 12.8-18.3%) TLC- 3400/mm^3 ^ (Adult males: 5000-10000/uL Adult females: 4500-11000/uL Children: 5000-10000/uL New born: 9000-30000/uL) · Platelet count- 85000/mm^3 ^ (Adult males: 1.35-3.17L/mcL Adult females: 1.57-3.71L/mcL Children: 2.5-4.5L/mcL New born: 1.5-4.5L/mcL) · PBS- Normocytic normochromic anemia, anisopoikilocytosis with leukothrombocytopenia
LIVER FUNCTION TEST	Total bilirubin- 1.2 mg/dl (Adults: 0.3-1.2mg/dl Paediatric: 0-1mg/dl) Bilirubin direct- 0.2 mg/dl (<0.2 mg/dl) Bilirubin indirect- 1 mg/dl (0.2-0.8 mg/dl) AST – 52 U/L (Males: <50 U/L Females: <35 U/L) ALT – 55U/L (Males: <50U/L Females: <35U/L) Albumin – 3.39 g/dl (3.5-5.2g/dl) ALP – 232 U/L (Adults: 38-126 U/L Paediatrics: 44-147 U/L) PT – 14 sec (Normal range: 11-13.5s) INR – 1 (Normal range: 0.8-1.1)
RENAL FUNCTION TEST	Urea – 23 mg/dl (Normal range: 17-43 mg/dl) Creatinine – 0.62 mg/dl (Males: 0.7-1.18 mg/dl Females: 0.55-1.02 mg/dl Paediatric: 0.5-1.0 mg/dl) Uric acid – 2.2 mg/dl (Males: 3.5-7.2 mg/dl Females: 2.6-6 mg/dl Paediatric: 2.5-5.5 mg/dl)
VIRAL MARKERS	· HIV, Anti- HCV, HBsAg negative
SERUM CERULOPASMIN	Patient’s value - <9.25 mg/dl (Normal range – 20 - 60 mg/dl)
24 HOUR URINE COPPER	Patient’s value – 292.47 µg/L (Normal range – 2-80 µg/dl) Patient’s value - 438.71 µg/day (Normal range – 3-50 µg/day)

**Figure 3:**
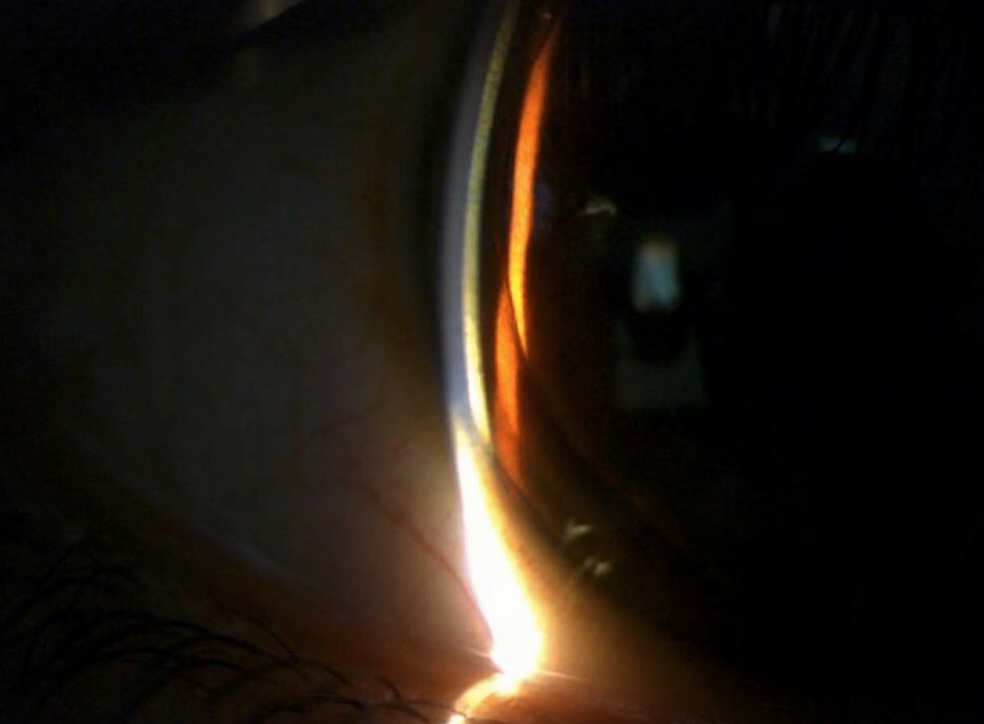
Kayser Fleischer rings in right cornea under slit lamp microscope

**Figure 4:**
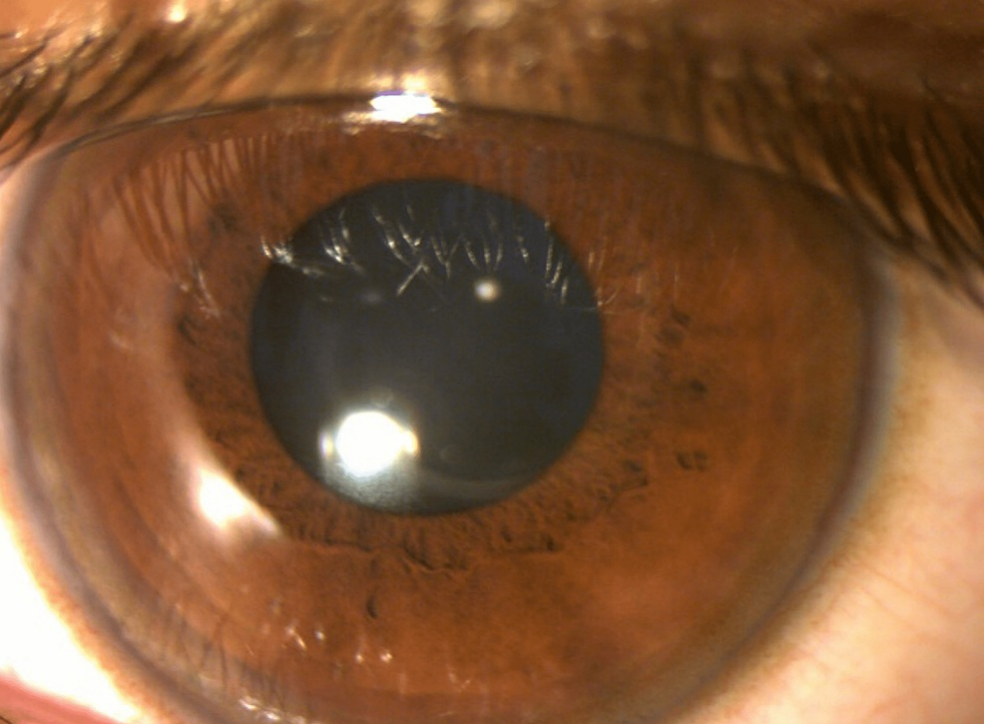
Kayser Fleischer ring in left cornea

Kayser Fleischer rings were discovered during a slit lamp examination in both corneas (Figures 3, 4).

**Figure 5:**
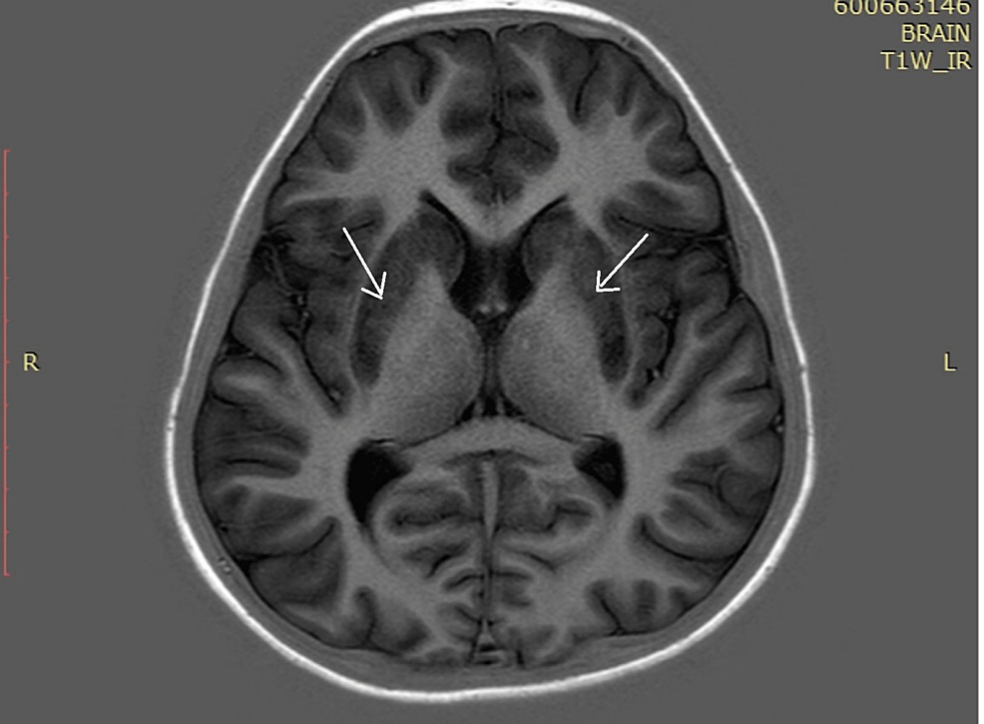
MRI T1-weighted sequence showing diffuse and symmetric hyperintensity of bilateral caudate and lentiform nuclei MRI: Magnetic Resonance Imaging

**Figure 6:**
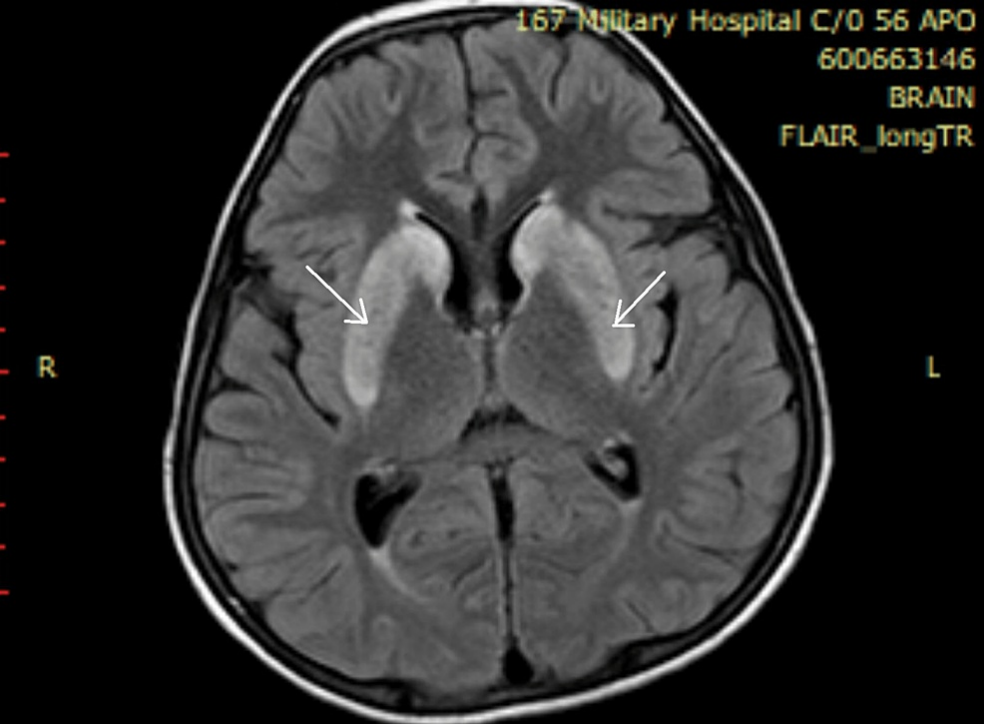
Hyperintensity in basal ganglia as seen on MRI FLAIR MRI: Magnetic Resonance Imaging FLAIR: Fluid Attenuated Inversion Recovery

Ultrasonography (USG) abdomen revealed hepatomegaly (liver span 14.8 cm), splenomegaly (16 cm) with coarse echotexture of the liver with no evidence of ascites or portal hypertension. Magnetic resonance imaging (MRI) of the brain revealed diffuse and symmetric hyperintensity of bilateral caudate and lentiform nuclei on T1, T2 and fluid attenuated inversion recovery (FLAIR) sequences, which are consistent with neuro-parenchymal changes of Wilson’s disease (Figure 5). Hyperintensity of the basal ganglia was also observed (Figure 6).

Using these parameters, patient was diagnosed with Wilson’s disease. He was restricted to a low copper diet and started on D-penicillamine 500 mg twice daily.

## Discussion

Wilson's illness is difficult to diagnose and treat due to its diverse clinical presentation, especially in environments with few resources. Due to a low index of suspicion for the condition, Wilson's disease cases are significantly underreported, which delays diagnosis and has a negative impact on the course of treatment [3]. A mutation in the long arm of chromosome 13's ATP7B copper transporting ATPase gene is the disease's etiological factor [4]. The age at which symptoms first appear might range from five to 74 years, but symptoms rarely appear before the age of five. Although it can have hematological, skeletal, or renal symptoms, it typically manifests as hepatic (particularly in the early decades of life) or neuropsychiatric traits (ordinarily in individual's second or third decades of life), although it can also present with hematological, skeletal, or renal signs [5]. Neuropsychiatric symptoms made up about 80% of the first presentation in a research on a large Indian case series, while hepatic symptoms made over 20% [2]. Most frequently, family screening is used to diagnose asymptomatic people. Dysarthria, dysphagia, excessive salivation, mood/behavior changes like depression and irritability, incoordination (like declining handwriting), declining academic performance, resting and intention tremors, gait disturbance, dystonia, rigidity, and other symptoms are among the most common neuropsychiatric symptoms. Risus sardonicus, stroke-like symptoms, and a mask-like face. Increased serum transaminases, acute hepatitis, hepatomegaly, fatty liver, acute liver failure with hemolysis, portal hypertension (esophageal varices, splenomegaly, low platelet count), and decompensated cirrhosis with ascites are examples of common hepatic symptoms. Other systemic symptoms include hemolytic anemia, renal tubular failure and nephrolithiasis, rickets, Kayser-Fleischer rings in the cornea, and other conditions [6]. In our situation, both neuropsychiatric and hepatic characteristics presented in a mixed but uncommon way. However, a thorough clinical examination together with research helped in the early detection of this condition and the establishment of treatment. Using the criteria established at the 8th International Wilson's Disease Meeting, Leipzig, 2001, our patient fits the criteria for this illness. There are numerous therapy options available including copper chelating agents (D-penicillamine) [6].

## Conclusions

Wilson's illness is a rare condition, thus a missed diagnosis is likely. Additionally, it's crucial to remind patients not to quit their therapy. The patient exhibited a number of peculiar characteristics. Our patient was released with medical therapy and a follow-up appointment was set for three months. Parents received advice on the nature of the illness, dietary restrictions, the significance of additional vaccinations, and drug side effects. This is a curable condition that requires clinical focus and several investigations to accurately diagnose and reduce mortality and morbidity from it, particularly in juvenile populations with diverse liver issue presentations. 
