# Correction: A Case of Megaspleen With Micrographism

**DOI:** 10.7759/cureus.c109

**Published:** 2023-04-19

**Authors:** Subrahmanyan VR, Deepu V Joy, Sweta Sahu, Anagha SK, Abhishek LNU, Vempati Roopeessh, Prerna Chandra, Vishal Venugopal, Sourav Bansal, Rishman Tandi

**Affiliations:** 1 Internal Medicine Pediatrics, Armed Forces Medical College, Pune, IND; 2 Internal Medicine, 167 Military Hospital, Pathankot, IND; 3 Internal Medicine, JJM Medical College, Davanagere, IND; 4 Internal Medicine, Government Medical College, Thiruvananthapuram, IND; 5 Medicine, Government Medical College, Amritsar, IND; 6 Internal Medicine, Gandhi Medical College and Hospital, Hyderabad, IND; 7 Internal Medicine, Deccan College of Medical Sciences, Hyderabad, IND; 8 Internal Medicine, Bhaarath Medical College and Hospital, Chennai, IND

This article has been corrected at the request of the authors to add Subrahmanyam VR and Deepu V. Joy as the first and second authors, respectively. Their omission from the original submission was an oversight on the part of the submitting author.

